# Tumor-infiltrating lymphocytes provides recent survival information for early-stage HER2-low-positive breast cancer: a large cohort retrospective study

**DOI:** 10.3389/fonc.2023.1148228

**Published:** 2023-06-20

**Authors:** Teng Sun, Tong Wang, Xiangjun Li, Haibo Wang, Yan Mao

**Affiliations:** ^1^Department of Surgery, School of Clinical Medicine, Qingdao University, Qingdao, China; ^2^Breast Disease Center, The Affiliated Hospital of Qingdao University, Qingdao, China; ^3^Department of Statistics, School of Public Health, Qingdao University, Qingdao, China

**Keywords:** tumor infiltrating lymphocytes (TILs), breast cancer, HER2-low-positive, tumor microenvironment (TME), clinical outcome assessment

## Abstract

**Purpose:**

It has been reported that breast cancer (BC) with low expression of human epidermal growth factor receptor 2 (HER2) might be a distinct subtype of BC. However, the prognostic effect of low HER2 expression on BC patients remains controversial. We aim to conduct this single-institution retrospective analysis to assess HER2-low-positive BC outcomes in Chinese women and the prognostic role of TILs in HER2-low-positive early-stage BC.

**Method:**

We retrospectively enrolled 1,763 BC patients treated in a single institution from 2017 to 2018. TILs are regarded as continuous variables and are divided into low TILs (≤10%) and high TILs (>10%) for statistical analysis. Univariate and multivariable Cox proportional hazards regression models were used to test the associations between TILs and disease-free survival (DFS) with adjustment for clinicopathologic characteristics.

**Result:**

High TIL levels (>10%) were associated with tumor size (>2 cm, p = 0.042), age at diagnosis (p = 0.005), Ki-67 index (>25%; p <0.001), HR (hormone receptor) status (positive, p <0.001), advanced pathological stage (p = 0.043), subtype (p <0.001), and HER2 status (p <0.001). The Kaplan−Meier analysis indicated that no significant difference in DFS (p = 0.83) could be found between HER2-positive, HER2-low-positive, and HER2-0 BC. The DFS of HER2-low-positive BC and HER2-nonamplified BC with high levels of TILs was statistically better than that of patients with low levels of TILs (p = 0.015; p = 0.047). In HER2-low-positive BC patients with high TIL levels (>10%), DFS was significantly improved in both the univariate (HR = 0.44, 95% CI 0.22–0.87, P = 0.018) and multivariate (HR = 0.47, 95% CI 0.23–0.95, P = 0.035) Cox models. For further subgroup analysis, HR (+)/HER2-low-positive BC with high TIL (>10%) levels was associated with improved DFS in both the univariate (HR = 0.41, 95% CI 0.19–0.90, P = 0.025) and multivariate (HR = 0.42, 95% CI 0.19–0.93, P = 0.032) Cox models. The HR (−)/HER2-0 BC with high TIL (>10%) level was not statistically significant in the univariate Cox model, but it was statistically significant in the multivariate (HR = 0.16, 95% CI 0.28–0.96, P = 0.045) Cox model.

**Conclusion:**

Among early-stage BC, no significant survival difference could be found between the HER2-positive, HER2-low-positive, and HER2-0 cohorts. High levels of TILs were significantly associated with improved DFS in HER2-low-positive patients, especially in the HR (+)/HER2-low-positive subtype.

## Introduction

Human epidermal growth factor receptor 2 (HER2) is a transmembrane glycoprotein with tyrosine kinase activity on the cell surface. It activates tumor cell proliferation signaling pathways upon dimerization and is involved in tumor proliferation and migration (1). With the improvement of detection technology and the use of molecularly targeted drugs, the HER2 low expression subtype of BC is gradually coming into the limelight, and its clinical and molecular phenotypic characteristics are gradually becoming the focus of attention. Several novel quantitative assays are under development to offer a more standardized, objective, and automated assessment of HER2, such as the automated quantitative analysis technology, the HERmark technology, and the quantitative IHC technology (2). According to prevailing HER2 testing guidelines, the most commonly used criterion is the combination of IHC and ISH results; either IHC 3+ or IHC 2+/FISH (+) is defined as HER2 positive (3). However, 45% to 55% of these patients with BC defined as HER2 negative actually have HER2 low expression (IHC 1+ or 2+, but FISH negative) (4), and recent reports show that no survival benefit could be obtained from the combination of HER2-targeted therapeutics with chemotherapy among women with HER2 low-positive BC (5–7). However, two HER2-directed antibody−drug conjugates (ADCs) with the chemotherapeutics trastuzumab deruxtecan (T-DXd) and trastuzumab duocarmazine (SYD985) have shown very promising therapeutic activity in patients with HER2-low-positive BC (8–10). A phase III trial of T-DXd involving patients with HER2-low metastatic BC recently concluded that trastuzumab deruxtecan resulted in significantly longer progression-free and overall survival than the physician’s choice of chemotherapy (i.e., NCT03734029/DESTINY-Breast04) (8).

In recent years, a series of prospective clinical trials have suggested that BC with low expression of HER2 might be a distinct subgroup of BC, which is different from BC with HER2 negative and HER2 positive in terms of biological characteristics and prognosis. Denkert et al.’s pooled analysis of four prospective clinical trials included 2,310 patients with HER2-non-amplified primary BC that were treated with neoadjuvant combination chemotherapy. The HER2-low-positive tumors had a significantly lower pathological complete response rate than HER2-0 tumors, but the 3-year overall survival of HER2-low-positive BC was significantly better than HER2-0 BC (11). However, in a study of 1,150 early-stage BC patients by Rossi et al., HER2-low-positive (ICH 2+/FISH−) status is an adverse prognostic factor in patients with operable BC (12). Consistently, Eggemann et al. demonstrated that the HER2 (2+) status is an unfavorable prognostic factor for the survival of patients with HR (+) BC, while the authors believed that the impact of anti-HER2 therapy in this group of patients should be evaluated (13). Therefore, the prognostic effect of low HER2 expression on BC patients remains controversial. There are few published data on HER2-low-positive and HER2-0 BC survival outcomes in Asian women. In addition, several studies have shown that TILs are an important indicator of the outcome of triple-negative breast cancer (TNBC) and HER2-positive BC (4, 14–16), but the prognostic effect and clinical significance of TILs in HER2-low-positive BC have not been reported. Therefore, we conducted this single-institution retrospective analysis to assess the HER2-low-positive BC outcomes in Chinese women and the prognostic role of TILs in HER2-low-positive early-stage BC.

## Materials and methods

### Patient enrolment and information collection

**Figure 1:**
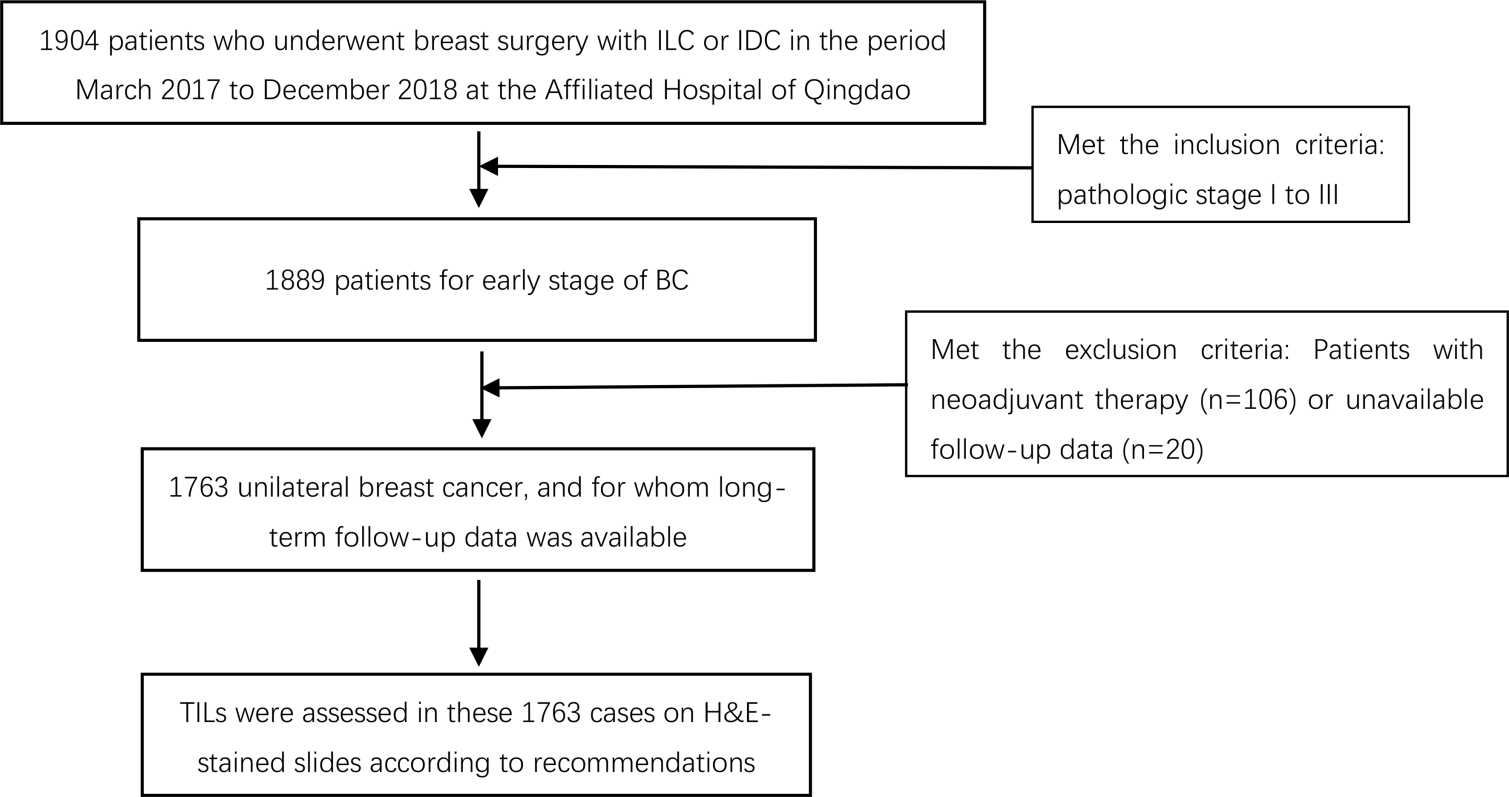
Work flow of the case using data from a mono-institutional study.

We investigated 1,904 patients with BC who underwent surgery from March 2017 to December 2018 at the Affiliated Hospital of Qingdao University, Qingdao, China. For this study, the inclusion criteria for the patients were (1) female and pathologically diagnosed with invasive lobular carcinoma (ILC) or invasive ductal carcinoma (IDC) and (2) pathologic stage I to III based on the AJCC 8th TNM staging system (17). The exclusion criteria included the following (1): diagnosis of ductal carcinoma *in situ* (DCIS) or DCIS with microinvasion (DCIS−Mi); (2) simultaneous bilateral BC (interval between diagnosis of tumors on both sides <6 months); (3) unavailable follow-up data; and (4) receiving preoperative neoadjuvant therapy (including neoadjuvant chemotherapy and neoadjuvant endocrine therapy). After the inclusion and exclusion criteria were applied, a total of 1,763 patients were included in this study (**Figure 1**). We retrospectively reviewed clinicopathological features, including age, Ki-67 labeling index, tumor size, lymph node status, ER, PR and HER2 status, pathologic stage, and stromal TIL level, from clinical medical records and pathology databases. Follow-up information was obtained from the hospital information system or through telephone interviews with the patients or relatives. DFS, as the primary end point of the study, was defined as the time interval from surgery to disease progression (including ipsilateral or contralateral BC recurrence, local/distant metastasis) or death in patients with BC. The follow-up ended in July 2022.

### Breast cancer subtypes

BC tumors were classified into subtypes TNBC, HER2-positive, and luminal breast cancer (LBC), which were determined by ER, PR, HER2, and Ki-67 based on IHC and FISH from the patients’ pathologic reports. ER and PR ≥1% were defined as positive, and for HER2, an immunohistochemistry (IHC) score of 3+ or amplification of fluorescence *in situ* hybridization (FISH) was considered positive according to the American Society for Clinical Oncology (ASCO)/College of American Pathologists (CAP) guidelines. HER2 nonamplified BC was classified as HER2-low-positive (IHC 1+ or IHC 2+ and FISH negative) and HER2-0 (IHC-0). Because a Ki-67 threshold of at least 25% of immunostained cells has been shown to provide the most powerful outcome prognostication, patients with Ki-67 scores above this threshold were considered positive (18).

### Assessment of stromal TILs

**Figure 2:**
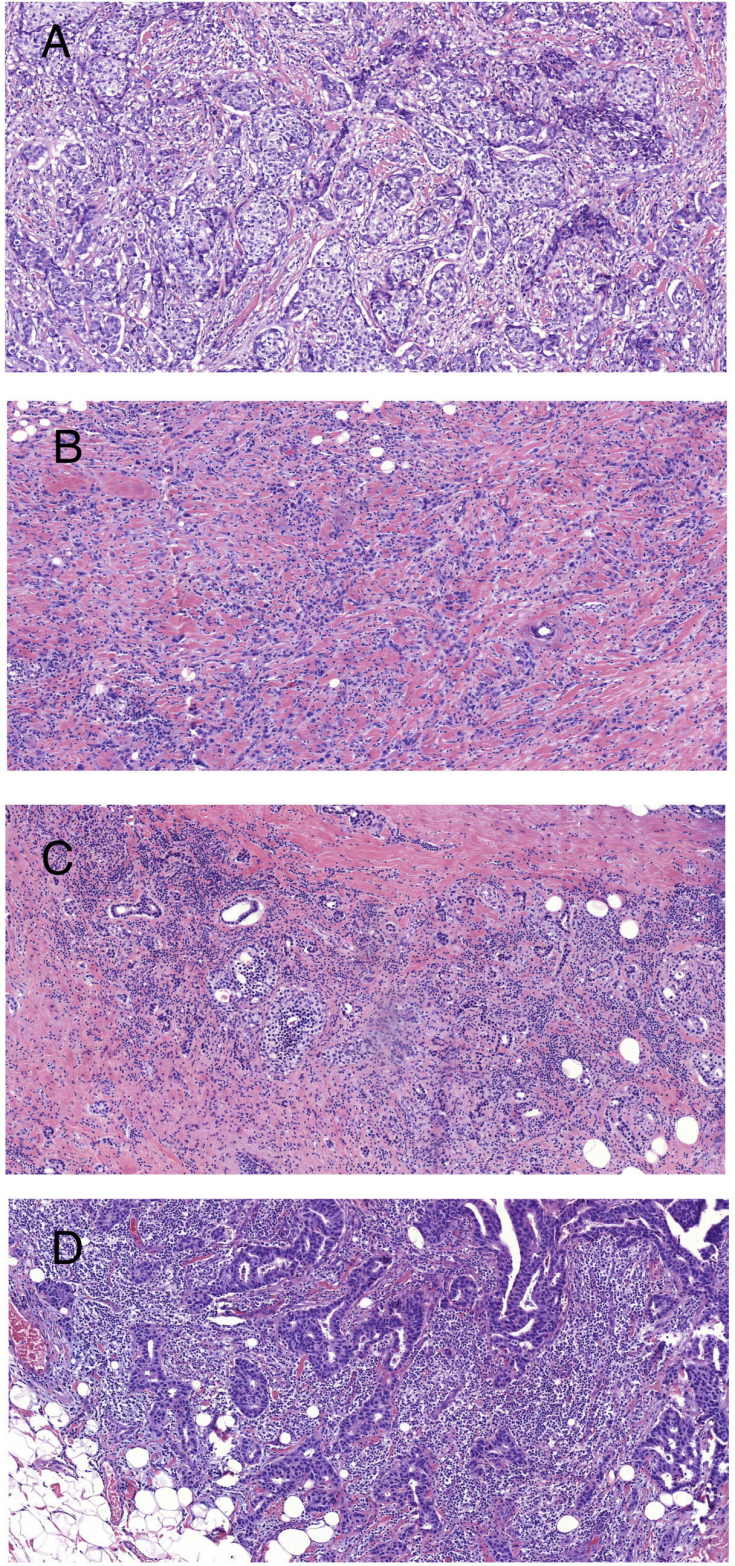
Representative images of BC with different densities of stromal TILs (H&E staining at ×100 magnification). **(A)** TILs = 5%; **(B)** TILs = 10%; **(C)** TILs = 50%; and **(D)** TILs = 90%.

In accordance with the recommendations of the international TILs Working Group, we evaluated stromal TILs with whole sections of hematoxylin and eosin (H&E)-stained slides (4). The stromal area was defined as the specialized stroma surrounding the invasive tumor cells. Brief evaluation principles: The specific percentage images of TILs provided in the reference guidelines were used for image reference assessment. TILs are the percentage of lymphocyte area in the stromal area of tumors in all stromal areas. The assessment of lymphocytes included all monocytes infiltrating the stroma within the invasive margin of BC (including lymphocytes and plasma cells, excluding lobulated nuclear cells). **Figure 2** illustrates samples of BC with different densities of TILs. The TIL level was divided by 10% as the cut-off value, which was defined using the criteria suggested by Denkert et al. (19) and was consistent with previous studies (20, 21).

### Statistical analysis

Statistical analyses were conducted with SPSS version 26.0 and R version 3.6.1. Univariate and multivariate Cox proportional hazards regression models were used to assess differences in DFS across the different groups defined by TILs. We used the Kaplan–Meier curve to analyze DFS and the log-rank test for comparison. Univariate and multivariate Cox regression models and 95% confidence intervals (CIs) were used to analyze the significance of the influence of TILs on DFS in BC patients. All tests were two-sided, and a P-value of <0.05 was considered statistically significant.

## Results

### Patient characteristics

There were a total of 1,763 patients who were identified between March 2017 and December 2018 in our study, of whom 429 were HER2-positive, 739 were HER2-low-positive, and 595 were HER2-0. The patients ranged in age from 26 to 88 years (average 56 years). The median follow-up time was 49 months (range 4–69 months). At the endpoint of the follow-up, 79 patients had progressed, of whom 20 were HER2-positive, 32 were HER2-low-positive, and 27 were HER2-0. When TILs were taken as a dichotomous variable with 10% as the bound value, higher TIL levels (>10%) were significantly associated with more aggressive clinicopathologic characteristics, such as larger tumor size (>2 cm, *p* = 0.042), age at diagnosis (*p* = 0.005), Ki-67 index (>25%; *p <*0.001), HR status (positive, *p <*0.001), advanced pathological stage (*p* = 0.043), subtype (*p <*0.001), and HER2 status (*p <*0.001). Additionally, no obvious differences were observed in lymph node status. **Table 1** lists more information about TILs with clinicopathological characteristics in the overall population.

**Table 1:** Associations between TILs and clinicopathological factors in the overall population.

	TILscat1		Total	*p*-value
	≤10%	>10%		
**Age**				0.005
≤55	328 (37.4%)	548 (62.6%)	876 (49.7%)	
>55	391 (44.1%)	496 (55.9%)	887 (50.3%)	
**ER**				<0.001
positive	607 (45.8%)	719 (54.2%)	1,326 (75.2%)	
negative	112 (25.6%)	325 (74.4%)	437 (24.8%)	
**PR**				<0.001
positive	575 (46.3%)	668 (53.7%)	1,243 (70.5%)	
negative	144 (27.7%)	376 (72.3%)	520 (29.5%)	
**Ki-67 index**				<0.001
≤25%	467 (53.7%)	402 (46.3%)	869 (49.3%)	
>25%	252 (28.2%)	642 (71.8%)	894 (50.7%)	
**Tumor size**				0.042
≤2 cm	427 (42.9%)	569 (57.1%)	996 (56.5%)	
>2 cm	292 (38.1%)	475 (61.9%)	767 (43.5%)	
**Lymph node status**				0.154
negative	422 (42.2%)	577 (57.8%)	999 (56.7%)	
positive	297 (38.9%)	467 (61.1%)	764 (43.3%)	
**pTNM stage**				0.043
I	302 (45.5%)	362 (54.5%)	664 (37.7%)	
II	311 (37.2%)	524 (62.8%)	835 (47.4%)	
III	106 (40.2%)	158 (59.8%)	264 (15.0%)	
**Relapse**				0.022
No	677 (40.2%)	1,007 (59.8%)	1,684 (95.5%)	
Yes	42 (53.2%)	37 (46.8%)	79 (4.5%)	
**Subtype**				<0.001
Luminal/HER2-	544 (48.7%)	574 (51.3%)	1,118 (63.4%)	
Luminal/HER2+	68 (30.8%)	153 (69.2%)	221 (12.5%)	
HR−/HER2+	51 (24.5%)	157 (75.5%)	208 (11.8%)	
TNBC	56 (25.9%)	160 (74.1%)	216 (12.3%)	
**HER2 status**				<0.001
HER2-positive	119 (27.7%)	310 (72.3%)	429 (24.3%)	
HER2-low-positive	295 (39.9%)	444 (60.1%)	739 (41.9%)	
HER2-0	305 (51.3%)	290 (48.7%)	595 (33.7%)	

### Survival analysis

**Figure 3:**
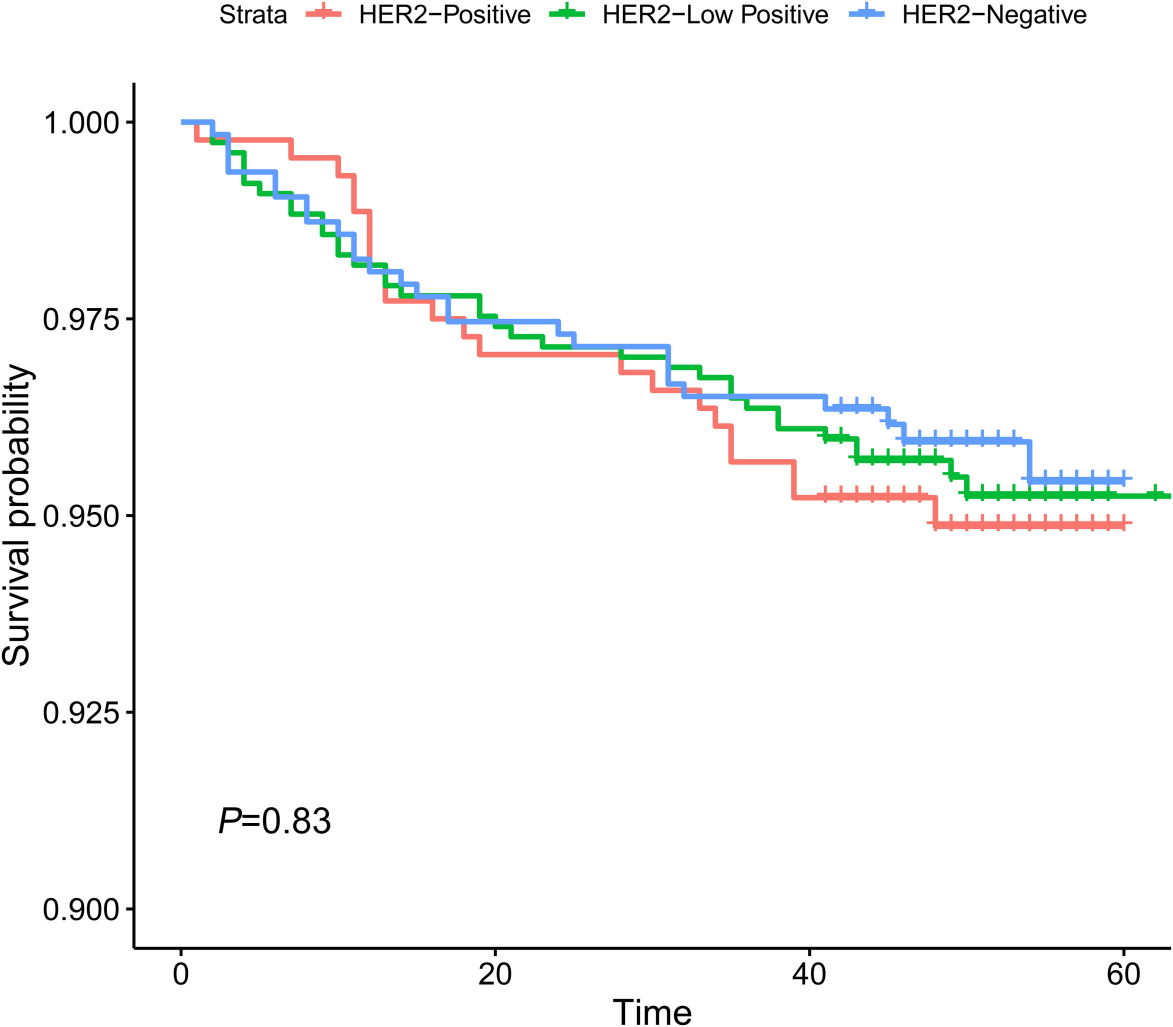
K–M analysis of DFS according to HER-2 expression status.

**Figure 4:**
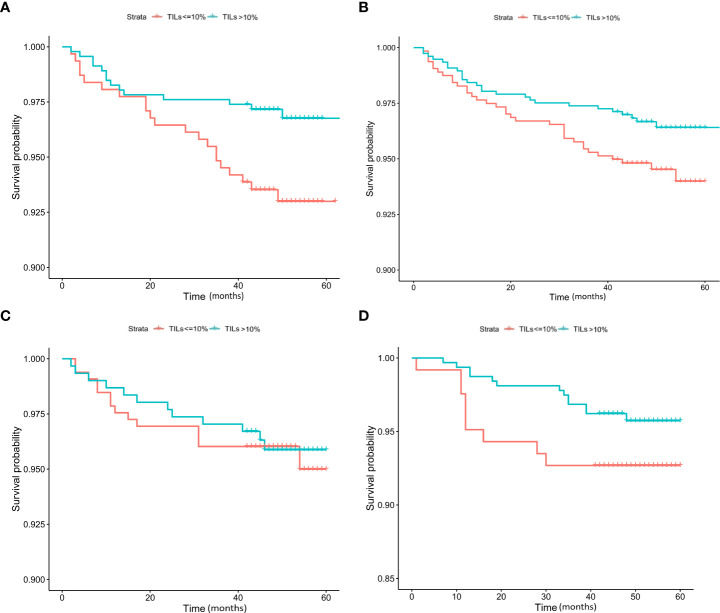
K–M analysis of DFS according to TIL level in the HER2-low-positive **(A)**, HER2-nonamplified **(B)**, HER2-0 **(C)**, and HER2-positive **(D)** subgroups.

Kaplan−Meier analysis indicated that no significant difference in DFS (p = 0.83) could be found between HER2-positive, HER2-low-positive, and HER2-0 BC (**Figure 3**). Several studies have shown that TILs are an important indicator of the outcome of BC patients (4, 14–16). Therefore, we then conducted a survival analysis according to TIL levels (≤10% vs. >10%). Our results showed that the DFS of HER2-low-positive BC and HER2-nonamplified BC with high levels of TILs was statistically better than that of HER2-low-positive BC, and HER2-nonamplified BC presented low levels of TILs (p = 0.015, **Figure 4A**; p = 0.047, **Figure 4B**). No significant difference in DFS was observed according to the TIL levels of the HER2-0 (p = 0.81, **Figure 4C**) and HER2 (p = 0.15, **Figure 4D**)-positive subgroups.

### Analysis in subgroups

Our analysis found that a high level of TILs not only differed significantly among each subtype of patient but was also closely associated with HER2 expression. Based on this finding, a further analysis of the prognostic effect of TILs on HER2 expression status was conducted. We found that in HER2-low-positive BC patients with high TIL levels (>10%), DFS was significantly improved in both the univariate (HR = 0.44, 95% CI 0.22–0.87, P = 0.018) and multivariate (HR = 0.47, 95% CI 0.23–0.95, P = 0.035) Cox models. Compared to low TIL levels (≤10%), the HER2-low-positive BC patients with high TIL levels (>10%) had a 53% reduced risk of recurrence and metastasis. In the HER2-0 BC patients with high TIL levels (>10%), DFS was not statistically significant in the univariate and multivariate Cox models. Because TIL levels were associated with HR status, we then constructed Cox models according to HR status (negative vs. positive). HR (+)/HER2-low-positive BC with high TIL (>10%) levels was associated with improved DFS in both the univariate (HR = 0.41, 95% CI 0.19–0.90, P = 0.025) and multivariate (HR = 0.42, 95% CI 0.19–0.93, P = 0.032) Cox models. Compared to low TIL levels (≤10%), the HR (+)/HER2-low-positive BC patients with high TIL levels (>10%) had a 58% reduced risk of recurrence and metastasis. HR (−)/HER2-0 BC with high TIL (>10%) levels was not statistically significant in the univariate Cox model; however, after correcting for confounding factors such as age, tumor size, pathological stage, lymph node status, and Ki-67 index, it was statistically significant in the multivariate (HR = 0.16, 95% CI 0.28–0.96, P = 0.045) Cox model. Compared to low TIL levels (≤10%), HR (−)/HER2-0 BC patients with high TIL levels (>10%) had an 84% reduced risk of recurrence and metastasis. **Table 2** lists more information about the univariate and multivariate Cox regression models for DFS in HER2-nonamplified BC.

**Table 2:** Univariate and multivariate cox regression model for DFS in HER2-nonamplified BC.

subgroups	Univariate			multivariate		
	HR	95%CI	P	HR	95%CI	P
**HER2-low-positive**	0.44	0.22–0.87	0.018*	0.47	0.23–0.95	0.035*
**HER2-0**	0.91	0.42–1.96	0.807	1.00	0.44–2.32	0.986
**HR (+)/HER2-low-positive**	0.41	0.19–0.90	0.025*	0.42	0.19–0.94	0.032*
**HR (−)/HER2-low-positive**	0.38	0.09–1.70	0.207	1.47	0.09–25.04	0.792
**HR (+)/HER2-0**	0.83	0.32–2.14	0.702	0.92	0.34–2.45	0.867
**HR (−)/HER2-0**	0.65	0.16–2.73	0.557	0.16	0.28–0.96	0.045*

* p<0.05.

## Discussion

HER2-targeted therapies have long been targeted at patients with HER2-overexpressing BC, but with the development and exploration of novel HER2-targeted antibody−drug conjugates (ADCs), patients with low HER2 expression BC can also benefit from them (9, 10). Due to the heterogeneity of HER2 expression, the impact of low HER2 expression on the prognosis of HER2-nonamplified primary BC is still controversial. Two previous studies reported that BC patients with HER2-low-positive (ICH 2+/FISH−) status had poorer outcomes than those with HER2-0 BC (12, 13). However, a recent pooled analysis of four prospective clinical trials including 2,310 patients showed that the 3-year overall survival of HER2-low-positive BC was significantly better than that of HER2-0 BC (11). In our study, no significant difference in DFS was found between HER2-0, HER2-low-positive, and HER2-positive BC. The discrepancy between these studies might be because the definition of HER2-low-positive BC was not identical, as two previously reported studies showed HER2-low-positive defined as (ICH 2+/FISH−), while a recent pooled analysis and our study defined HER2-low-positive as (IHC) 1+ or IHC2+/*in situ* hybridization negative. In addition, HR status, ethnicity, and follow-up duration also affect the outcomes. We thought that a more detailed classification of HER2 status and a longer follow-up duration would be helpful in future studies. Therefore, additional markers are needed to identify high-risk patients with HER2-nonamplified BC. Considering TILs as an important predictor of prognosis in BC patients (4, 14–16), we found that patients with higher TIL levels (>10%) in HER2-low-positive and HER2-nonamplified BC had a significant survival benefit compared with patients with lower levels of TILs (≤10%).

High levels of TILs appear to reflect favorable host antitumor immune response status, improve BC clinical outcomes, and obtain more benefit from neoadjuvant therapy, particularly in TNBC and HER2+ BC (4, 14). A total of 1,334 HER2-nonamplified BC patients with stromal tumor-infiltrating lymphocytes (sTILs) were identified at our institute. We found that high TIL levels were related to more aggressive clinicopathologic characteristics, such as larger tumor size, age at diagnosis, Ki-67 index, HR status, advanced pathological stage, subtype, and HER2 status. We further analyzed the association between TILs and clinical outcomes. In HER2-low-positive BC patients with high TIL levels, DFS was significantly improved in both the univariate and multivariate Cox models. Similarly, the TIL level in the HR (+) HER2 low expression group was associated with improved DFS in both the univariate and multivariate Cox models. In addition, HR (−)/HER2-0 BC with high TIL levels was not statistically significant in the univariate Cox model; however, after correcting for confounding factors, it was statistically significant in the multivariate Cox model.

It has been proposed that TILs, as an important component of the tumor microenvironment (TME) prior to treatment, can predict the response to therapy and prognosis (19, 22, 23). With the development of immuno-oncology, the interaction between tumor and immune cells may be an important factor in prognosis, and there is evidence that both chemotherapy and endocrine therapy may be associated with tumor-immune interactions. Montagna et al. reported that an increase in TILs was associated with shorter overall survival in hormone receptor-positive, HER2-negative metastatic BC (24). Zhang et al. performed a retrospective analysis and found that high TIL levels tended to have a worse DFS for luminal A BC (21). In contrast, Honda et al. found that in luminal/HER2− BC, high levels of Ki-67 expression and patients with an intermediate or high sTIL level had a better prognosis than those with a low sTIL level (25). In another cohort including 987 patients with ER+/HER2− breast tumors receiving adjuvant chemotherapy, high TILs were associated with better DDFS, and high Ki-67 expression remained significantly associated with TILs in a multivariate regression analysis (26). However, there are no published data on the prognostic value of TILs for the clinical outcome of early HER2-nonamplified BC. A previous study reported that hormone receptor status might be another factor impacting the survival outcome of BC patients (27). We conducted subgroup analysis according to HR status and found that higher TIL levels in the HER2-low-positive subgroup, especially in the HR (+)/HER2-low-positive subgroup, were associated with improved DFS in both the univariate and multivariate Cox models. Therefore, for early-stage HER2-nonamplified BC patients, TILs could provide additional clinical outcome information.

In recent years, research on TIL-mediated immunotherapy using the patient’s own highly heterogeneous TILs for precision targeting of tumor cells has developed rapidly (28). Immunotherapy using checkpoint inhibitors has been successfully applied to TNBC; however, highly heterogeneous BC tumors often lead to suboptimal results (29). The combination of TIL-ACT and anti-PD-1/PD-L1 therapy may have important implications for immunotherapy in BC (30). Recently, data from a phase II clinical study have been reported, and the study investigated the therapeutic efficacy in a pilot trial of tumor-infiltrating lymphocytes (TILs) with a short course of pembrolizumab in patients with metastatic BC. Ultimately, of the six patients who participated in the treatment, objective tumor regression was noted in three LBC patients, including one with a complete response (ongoing over 5.5 years) and two with partial responses (6 and 10 months) (31). Adoptive transfer of TILs is a highly personalized experimental option for BC patients that has been shown to be capable of mediating objective responses in the current trial and deserves further study.

There are a few limitations to our study that need to be considered. Although all patients received standardized treatment for BC as recommended by the guidelines, treatment data on compliance and duration of endocrine therapy and chemotherapy were not documented, which may affect survival analysis in BC patients. Second, genomic information could not be available for the included patients and is closely related to the impact of survival on HER2-low expression among HER2-nonamplified BC (32). Last, due to the better prognosis of BC patients, extended follow-up end points may be more conducive to an accurate and comprehensive analysis of the prognostic status of the population. However, in terms of innovation, our study is the first to assess the prognostic role of TILs in HER2-nonamplified BC in an Asian population. Furthermore, multivariate analysis was used to control for confounding factors affecting outcomes. Considering the accuracy of TIL assessment, we strictly evaluated stromal TILs according to the guidelines recommended by the International Immuno-Oncology Biomarkers Working Group.

In summary, we found that stromal TIL levels showed significant prognostic value in HER2-nonamplified BC patients. Specifically, the DFS of HER2-low-positive BC and HER2-nonamplified BC with high levels of TILs was statistically better than that of those with low levels of TILs. High levels of TILs were significantly associated with improved DFS in HER2-low-positive patients, especially in patients with the HR (+)/HER2-low-positive subtype. The present study confirms that for early-stage HER2-nonamplified BC patients, TILs could provide additional clinical outcome information. Therefore, further investigation of the relationship between components of TILs and specific biomarkers of immunogenicity could better reveal prognosis and provide more information on HER2-nonamplified BC immunotherapy.

## Data availability statement

The raw data supporting the conclusions of this article will be made available by the authors, without undue reservation.

## Author contributions

TS contributed to conception, design of the study, organized the database and wrote the first draft of the manuscript. TW performed the statistical analysis. XL organized section of the database. TS, YM, and HW wrote sections of the manuscript. All authors contributed to the article and approved the submitted version.
